# Integrated convolution and self-attention for improving peptide toxicity prediction

**DOI:** 10.1093/bioinformatics/btae297

**Published:** 2024-05-02

**Authors:** Shihu Jiao, Xiucai Ye, Tetsuya Sakurai, Quan Zou, Ruijun Liu

**Affiliations:** Department of Computer Science, University of Tsukuba, Tsukuba 3058577, Japan; Department of Computer Science, University of Tsukuba, Tsukuba 3058577, Japan; Department of Computer Science, University of Tsukuba, Tsukuba 3058577, Japan; Institute of Fundamental and Frontier Sciences, University of Electronic Science and Technology of China, Chengdu 610054, China; Yangtze Delta Region Institute (Quzhou), University of Electronic Science and Technology of China, Quzhou 324000, China; School of Software, Beihang University, Beijing 100191, China

## Abstract

**Motivation:**

Peptides are promising agents for the treatment of a variety of diseases due to their specificity and efficacy. However, the development of peptide-based drugs is often hindered by the potential toxicity of peptides, which poses a significant barrier to their clinical application. Traditional experimental methods for evaluating peptide toxicity are time-consuming and costly, making the development process inefficient. Therefore, there is an urgent need for computational tools specifically designed to predict peptide toxicity accurately and rapidly, facilitating the identification of safe peptide candidates for drug development.

**Results:**

We provide here a novel computational approach, CAPTP, which leverages the power of convolutional and self-attention to enhance the prediction of peptide toxicity from amino acid sequences. CAPTP demonstrates outstanding performance, achieving a Matthews correlation coefficient of approximately 0.82 in both cross-validation settings and on independent test datasets. This performance surpasses that of existing state-of-the-art peptide toxicity predictors. Importantly, CAPTP maintains its robustness and generalizability even when dealing with data imbalances. Further analysis by CAPTP reveals that certain sequential patterns, particularly in the head and central regions of peptides, are crucial in determining their toxicity. This insight can significantly inform and guide the design of safer peptide drugs.

**Availability and implementation:**

The source code for CAPTP is freely available at https://github.com/jiaoshihu/CAPTP.

## 1 Introduction

Peptides, comprising sequences of amino acids connected by peptide bonds, are multifaceted organic molecules with lengths ranging from a few units to numerous units (Albericio and Kruger 2012, Apostolopoulos *et al.* 2021, Guntuboina *et al.* 2023, Zulfiqar *et al.* 2023). More than 7000 peptides have been identified to date, many of which have unique therapeutic properties, such as anticancer peptides (Chiangjong *et al.* 2020), antiviral peptides (Vilas Boas *et al.* 2019), and antimicrobial peptides (Lei *et al.* 2019), etc. In over a century, the Food and Drug Administration has approved more than 80 peptides, with approximately 400 oligopeptides presently under evaluation, revealing a promising future for peptides as effective clinically viable drugs (Cheng *et al.* 2021, Ni *et al.* 2022). Advances in peptide chemistry, molecular biology, and peptide delivery technology have progressively broadened the application of therapeutic peptides. The intrinsic chemical properties of these materials, such as versatility, biochemical diversity, and multifunctionality, extend their impact beyond pharmaceuticals to diagnostic, cosmeceutical, and biomedical research (Zhang *et al.* 2018, Ledwoń *et al.* 2021, Saw *et al.* 2021, Robles-Loaiza *et al.* 2022, Liu *et al.* 2023b, Wang *et al.* 2023, Li *et al.* 2024).

Drug development involves complex and critical steps, and only effective and non-toxic pharmaceuticals can be approved. Drug toxicity remains a persistent problem, and peptides are no exception (Blomme and Will 2016, Khan *et al.* 2018, Wang *et al.* 2023). Substantial efforts have been devoted to predicting and optimizing peptides; however, traditional toxicity assessment still largely depends on laborious and cost-intensive *in vivo* assessment (Sharma *et al.* 2022). Recently, many highly accurate and cost-effective machine learning techniques have been developed to predict protein toxicity (Jain and Kihara 2019, Sharma *et al.* 2022, Shi *et al.* 2022, Morozov *et al.* 2023, Song *et al.* 2023). While peptides and proteins share the same basic components (i.e. amino acids), there are still some differences in sequence length and structure (Jiang *et al.* 2023). Therefore, it is necessary to develop methods specifically targeted at peptide toxicity prediction. To our knowledge, there are only two machine learning models specifically designed for peptide toxicity prediction: ATSE and ToxIBTL (Wei *et al.* 2021, 2022). However, neither approach takes into account the data imbalance issues inherent in peptide toxicity prediction, often resulting in suboptimal overall performance. They use under-sampling to create balanced datasets, but this approach fails to fully utilize the majority of the samples. Furthermore, some identical sequences have annotation conflicts in different articles due to the inherent variability in experimental data. This can lead to decreased model performance, impede fair comparisons among models, and potentially result in overfitting and reduced trust in model reliability. Consequently, there is an urgent need for a well-prepared and reliable dataset for peptide toxicity prediction.

**Figure 1.**
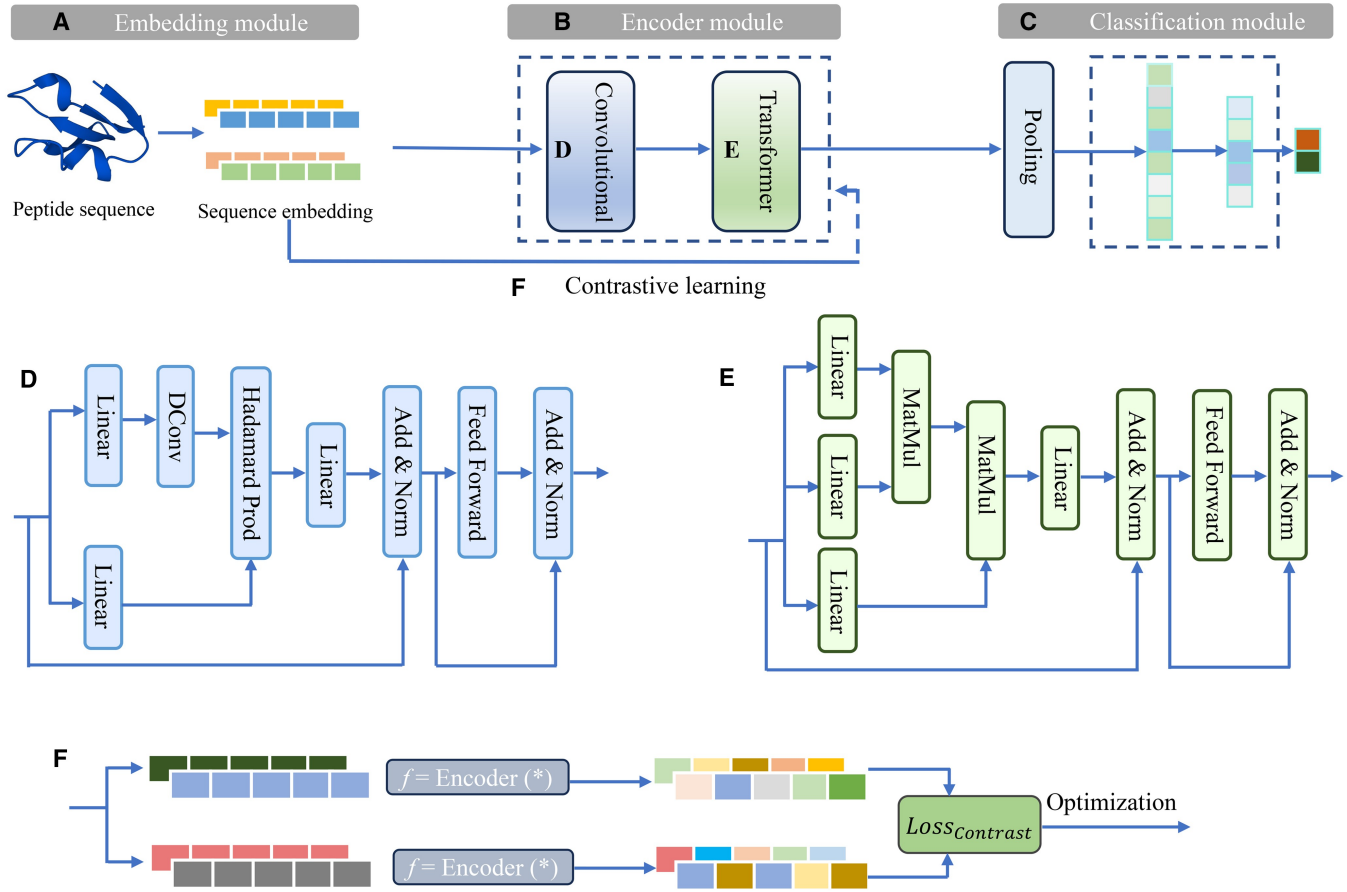
Overall architecture of CAPTP for peptide toxicity prediction.

The exciting advances in the natural language processing field, especially the rise of Transformer, have shed light on modeling peptide sequences due to the numerous similarities between them (Iuchi *et al.* 2021, Li *et al.* 2021, Ofer *et al.* 2021, Chen *et al.* 2023, Li and Liu 2023, Liu *et al.* 2023a, 2023b). Specifically, the self-attention mechanism in Transformer enables it to address long-range dependencies and capture comprehensive information across sequences (Wang *et al.* 2021). Despite these strengths, the features extracted by Transformer models tend to be generalized, lacking the specificity or sensitivity to a single or minor change in the sequence, an essential aspect of in-depth analysis of peptides at the amino acid level. Recently, many groups have explored the introduction of the concept of convolution into Transformer architecture to introduce soft inductive bias with better locality, thereby benefiting from the advantages of both architectures (d’Ascoli *et al.* 2021, Dai *et al.* 2021). In this study, by taking advantage of convolution and Transformer, we developed convolutional and attention-based peptide toxicity prediction (CAPTP), as illustrated in Fig. 1. CAPTP is an end-to-end model that predicts peptide toxicity using only amino acid sequences as the input with no additional features. Specifically, our model employs a novel encoder that combines convolutional modulation and self-attention to automatically learn representations of peptide sequences. Moreover, we used contrastive learning to alleviate the impact of data imbalance. Comparative experiments showed that our approach outperforms established protein and peptide toxicity benchmarks, such as CSM-Toxin (Morozov *et al.* 2023), ToxinPred2 (Sharma *et al.* 2022), and ToxIBTL (Wei *et al.* 2022). These findings position CAPTP as a valuable tool for the scientific community, enhancing the efficiency and accuracy of peptide toxicity screening and analysis. Using CAPTP, we quantitatively assessed the contributions of amino acids and identified important sequential characteristics. We found that both the head and mid regions of the peptide, as well as several specific sequential patterns, are strongly associated with peptide toxicity.

## 2 Materials and methods

### 2.1 Datasets

Here, a new dataset was constructed for predicting peptide toxicity, and the detailed workflow is described in Supplementary Fig. S1. First, we combined three datasets from previous studies on protein and peptide toxicity prediction, CSM-Toxin (Morozov *et al.* 2023), ToxinPred2 (Sharma *et al.* 2022), and ATSE (Wei *et al.* 2021), by selecting only samples shorter than 50 amino acids. It should be noted that we did not remove any duplicates at this stage. To obtain additional negative samples, we searched for manually annotated and reviewed non-toxic peptides in the UniProt (Consortium 2019) database (Release 2023_04) using the keywords “NOT KW-0800 AND NOT KW-0020 AND reviewed: true.” We excluded all sequences with non-standard amino acids or those exceeding 50 amino acids in length. These non-toxic peptides were then integrated into the previously consolidated dataset. During this integration, we removed 97 samples that had contradictory labels (Supplementary Table S1). After removing duplicates, we obtained 2491 toxic peptides (ToxPs) and 7053 non-toxic peptides (non-ToxPs). Next, we used CD-HIT (Fu *et al.* 2012) software with a similarity threshold of 0.9 to remove redundant sequences. The final dataset contained 2138 ToxPs and 5375 non-ToxPs, 85% of which were randomly selected to construct the training set, and the remaining 15% of the samples constituted the independent test set. Therefore, the training set consisted of 1818 ToxPs and 4569 non-ToxPs, while the independent test set consisted of 320 ToxPs and 806 non-ToxPs. The distribution of amino acids and sample sequence lengths within the training and testing datasets is summarized in Supplementary Fig. S2.

### 2.2 Model structure

As illustrated in Fig. 1, CAPTP is composed of four main modules: the embedding module, the encoder module, the prediction module, and the contrastive learning module. For any given peptide sequence, the model first combines token embeddings with position embeddings to generate the initial input representation. It is noteworthy that the adaptive embeddings are continuously updated via backpropagation. These embeddings are subsequently processed by the encoder layer, which uniquely integrates a convolutional encoder with a Transformer encoder, the core ideas of which are convolutional modulation and multi-head attention mechanism, respectively. This allows us to produce contextually enriched feature vectors for each amino acid, effectively capturing both local patterns and long-range dependencies. Max pooling is subsequently applied to the encoder’s feature matrix to represent the entire peptide sequence. The resulting peptide representation vector is then fed into the prediction module, which provides the probability distribution for specific classes. The contrastive learning module is employed to minimize the contrastive loss, ensuring that similar samples are brought closer and that dissimilar samples are pushed farther in the representation space, leading to more discriminative embeddings. The hyperparameters of the model are summarized in Supplementary Table S2. In the following section, we will describe several key components, including the convolutional modulation, the multi-head attention mechanism, and the contrastive learning module.

The Transformer encoder mainly contains a multi-head attention layer, a feedforward network, and a residual connection. The structure of the convolutional encoder is similar, as shown in Fig. 1D; however, the difference is that the self-attention layer is replaced with a convolutional modulation layer (Hou et al. 2022). In the convolutional modulation layer, we use the convolutional features to modulate the value V to simplify the traditional similarity score matrix in the self-attention mechanism. Specifically, for the embedding X of a peptide sequence of length L ($X\;\in R^{L\times d}$), where d is the embedding dimension, we employ a simple depthwise convolution with kernel size k and the Hadamard product to calculate the output Z of convolutional modulation as follows:

(1) $$\left\{\begin{array}{c} A=\mathrm{DCon}v_{k}(W_{1}X)\\ V=W_{2}X\\ Z=A⨀V \end{array}\right.$$

where $W_{1}$ and $W_{2}$ are weight matrices of two linear layers, ⨀ represents the Hadamard product, and $\mathrm{DCon}v_{k}$ denotes a depthwise convolution with kernel size k. This operation enables each position p in the sequence to correlate with all amino acids within the region of length k centered at p. The output for each sequence position is the weighted aggregate of all amino acids within the region of interest, promoting an enhanced local contextual understanding of the peptide sequence.

As mentioned above, the multi-head attention mechanism is the core idea of the Transformer. It has achieved numerous outstanding performances in many aspects of computational biology. Multi-head attention comprises several self-attention mechanisms to capture different types of internal correlations within the same sequence (as shown in Fig. 1E). For the output X ($X\;\in R^{L\times d}$) of the previous layer, self-attention first generates the key (K), query (Q), and value (V) through linear transformations (Q, *K*, and $V\;\in R^{L\times d_{k}}$, where $d_{k}$ represents the dimensionality of the transformed space). The final output is a weighted average of V, with weights determined by the similarity score S that measures relationships between amino acid pairs. For simplicity, we omit the scaling factor in this explanation and the mathematical description is as follows:

(2) $$\left\{\begin{array}{c} \mathrm{Attention}(X)=SV\\ S=\mathrm{Softmax}(QK^{T}) \end{array}\right.$$

Contrastive learning emphasizes distinguishing between similar and dissimilar sample pairs, potentially enhancing model robustness, especially for underrepresented classes in imbalanced datasets, while also providing a comprehensive feature representation that benefits minority classes (Zhang *et al.* 2022, Tao *et al.* 2024). Here, we use a contrastive learning module based on supervised learning that ensures that inputs from the same class are mapped closely in the representation space, while those from different classes are mapped farther away. Specifically, we collect a batch size of representation matrices from the encoder layer and construct a contrastive loss, $L_{\mathrm{contrast}}$, as one of our framework’s loss functions. This function calculates and optimizes the distance between the representations of sample pairs. For a pair of peptide representations $Z_{1}$ and $Z_{2}$ in a batch, the loss is defined as follows:

(3) $$\left\{\begin{array}{l} D\left(Z_{1},\;Z_{2}\right)=1-\mathrm{cosine}\left(Z_{1},{\;Z}_{2}\right)\\ L_{\mathrm{contrast}}(Z_{1},\;Z_{2},\;y)=\frac{1}{2}\left(1-y\right)D{\left(Z_{1},\;Z_{2}\right)}^{2}+{\frac{1}{2}y\left\{D_{\max}-D\left(Z_{1},\;Z_{2}\right)\right\}}^{3} \end{array}\right.$$

where $D\left(Z_{1},\;Z_{2}\right)$ measures the distance between $Z_{1}$ and $Z_{2}$. y is set to 1 when the pair of residues come from different classes, indicating that one sequence is toxic and the other is not. Conversely, *y* is 0 when both sequences belong to the same class. $D_{\max}$ is the maximum value of $D\left(Z_{1},\;Z_{2}\right)$ and is set to 1.5 in this study. Importantly, by assigning a power of three to the different-class pair, we indirectly steer the model’s focus toward the minority class. Finally, we combine the contrastive loss $L_{\mathrm{contrast}}$ with the binary cross-entropy loss to form the total loss function of our model. This composite loss function takes into account both the similarities and differences between sample pairs, as well as the accuracy of classification, thereby achieving a comprehensive optimization of the model. During the training process, we minimize this total loss to adjust the model parameters, aiming to enhance the model’s performance on the imbalanced dataset.

### 2.3 Evaluation metrics

In this work, the performance of all the models is evaluated using the following standard metrics: balanced accuracy (BACC), sensitivity (SN), specificity (SP), and Matthews correlation coefficient (MCC) (Yan *et al.* 2023, Zhu *et al.* 2023, Zou *et al.* 2023).

(4) SN=TPTP+FN*100%SP=TNTN+FP*100%BACC= 12(SN+SP)*100%MCC=(TP×TN)-(FP×FN)(TP+FP)×(TN+FN)×(TP+FN)×(TN+FP) 

where TP, TN, FP, and FN are true ToxPs, true non-ToxPs, false ToxPs, and false non-ToxPs, respectively. Given the imbalance in our dataset, we employ both the BACC and MCC to provide an overall assessment of the models’ performance. The SN reflects the ratio of toxic peptides accurately identified by the model, while the SP quantifies the accurately predicted non-toxic peptides. Additionally, the model’s overall performance is further quantified by the area under the receiver operating characteristic curve, commonly referred to as the area under the curve (AUC), providing a comprehensive measure of the model’s predictive capabilities. Collectively, higher values of these metrics indicate the superior predictive accuracy of the model.

## 3 Results

### 3.1 Exploration of the optimal architecture of CAPTP

We first evaluated the contribution of contrastive learning to CAPTP, through comparison to a variant without this module. The results are shown in Table 1. It is evident that the version of CAPTP with contrastive learning surpasses the model without this module. Specifically, there is a marked increase in three important metrics for measuring the model’s overall performance and ability: BACC, AUC, and MCC (23.0%, 10.6%, and 35.3%, respectively). Furthermore, when we focused on the SN, which is crucial for understanding true positive rates, our CAPTP showed a remarkable improvement, increasing from 41.63% to 89.56%, while the SP is only slightly reduced. These results confirm the efficacy of contrastive learning in improving the model’s predictive capability. One possible reason is that the incorporation of contrastive learning appears to help the model enhance the representation of minority classes, thereby offering a better solution to the imbalance challenge.

**Table 1.** Comparative analysis of CAPTP model performance across different architectural configurations using 5-fold cross-validation.

Hyperparameter	Value	BACC (%)	AUC	SN (%)	SP (%)	MCC
Contrastive learning	w	91.62	0.960	89.56	93.70	0.820
	w/o	68.64	0.854	41.63	95.64	0.467
Pooling method	CLS	90.20	0.953	85.04	95.36	0.812
	Max	91.62	0.960	89.56	93.70	0.820
	Mean	91.27	0.958	88.23	94.31	0.820
	Min	91.50	0.959	89.99	93.02	0.813
	Attention	91.14	0.955	87.19	95.10	0.824
Kernel size	*K* = 1	89.72	0.959	89.84	89.61	0.762
	*K* = 2	90.93	0.957	89.60	92.25	0.799
	*K* = 3	91.62	0.960	89.56	93.70	0.820
	*K* = 4	91.43	0.961	90.54	92.32	0.807
	*K* = 5	91.58	0.959	90.93	92.22	0.808

Having elucidated the significant impact of contrastive learning on CAPTP models, we now turn our attention to exploring the best way to represent the whole sequence. The standard practice is to add a special “[CLS]” token at the beginning of each sequence during data preprocessing. The output vector corresponding to this “[CLS]” token is then used as the learned feature vector for the entire sequence (Devlin *et al.* 2018). Here, we also compared the use of different pooling methods to process the output feature matrix of the encoder layer, including max, min, mean, and attention pooling. The output vector of the pooling layer or special token is input into the prediction module to compute the probability of the sequence being classified as ToxPs or non-ToxPs. As shown in Table 1, the max pooling outperforms the other four methods, with a BACC of 91.62%, an AUC of 0.960, and an MCC of 0.820. In contrast, the traditional “[CLS]” token representation lags behind, with lower BACC, AUC, and MCC values of 90.20%, 0.953, and 0.812, respectively. Interestingly, despite the theoretical efficacy of attention pooling, it does not exceed the efficacy of mean pooling, achieving 91.14% BACC, 0.955 AUC, and 0.824 MCC, respectively. Notably, even though attention pooling has a greater MCC than max pooling does, there is a significant discrepancy observed between the SN and SP. This finding suggests that attention pooling might not be as effective as max pooling in balancing the recognition of positive and negative classes.

Subsequently, our research further investigated the impact of the size of the convolution kernel in convolutional modulation on model performance. The kernel size directly determines the scope of the local context captured from the input, which directly impacts the quality and nature of the information fed into the Transformer encoder. A carefully chosen kernel size allows for an optimal balance between capturing detailed local features and broader contextual information, thus enhancing the overall performance. The results are also shown in Table 1. Here, a kernel size of 3 yielded optimal performance metrics. This kernel size facilitates the model’s capacity to incorporate relevant local information effectively. Based on all previous comprehensive analyses, we configured the CAPTP model with max pooling, a kernel size of 3, and integrated contrastive learning.

### 3.2 Comparative performance of CAPTP with existing models

**Figure 2.**
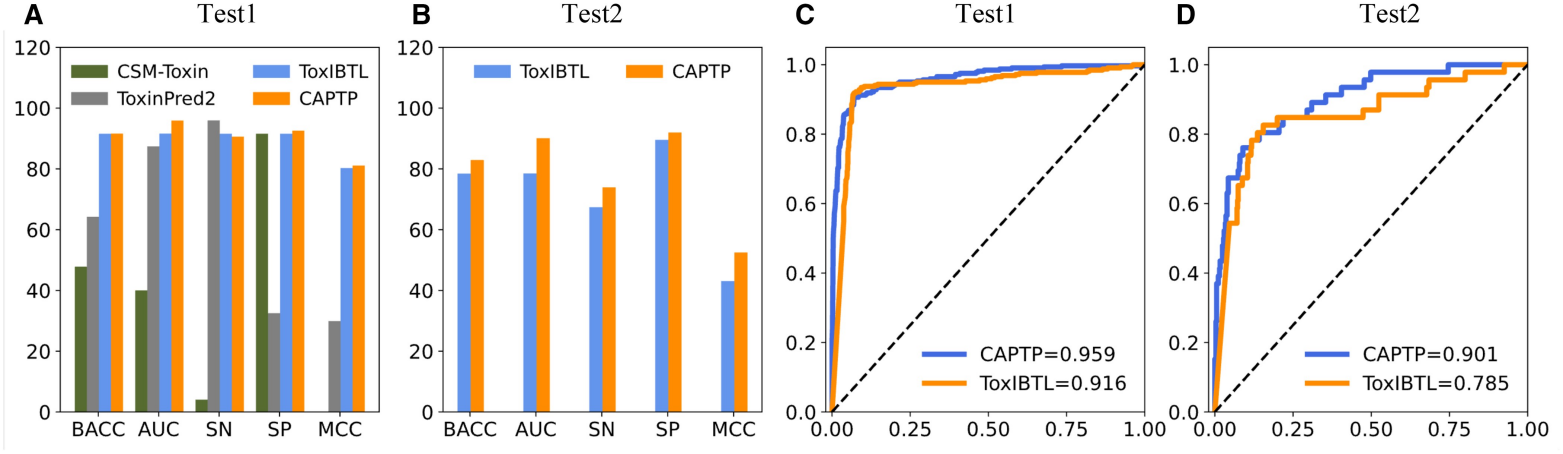
(A) and (B) show the performance comparisons of the existing methods. (C) and (D) show the ROC curves of the CAPTP and ToxIBTL for Test1 and Test2.

In this research, we benchmarked various computational models for predicting protein or peptide toxicity using an independent test dataset Test1. As shown in Fig. 2 and Table 2, CSM-Toxin and ToxinPred2, both designed for protein toxicity, present distinct profiles. CSM-Toxin offers high SP but low SN, resulting in limited overall performance, while ToxinPred2 provides high SN with lower SP, indicating a propensity to identify a broader range of peptides as toxic, potentially increasing false-positives. The ToxIBTL and our CAPTP models, both of which were specifically designed for peptide toxicity prediction, demonstrate closely matched performances, each exhibiting high BACC, with our model slightly leading. Both models share robust SNs and SPs above 90%, ensuring dependable predictions, while our model edges ahead with a slightly higher AUC and MCC.

**Table 2.** Performance comparison of CAPTP and the other state-of-the-art methods on the independent test sets.

Model	Dataset	BACC (%)	AUC	SN (%)	SP (%)	MCC
CSM-Toxin	Test1	47.81	0.400	4.06	91.56	−0.076
ToxinPred2	Test1	64.22	0.874	95.94	32.51	0.299
ToxIBTL	Test1	91.56	0.916	91.56	91.56	0.803
CAPTP	Test1	91.59	0.959	90.63	92.56	0.811
ToxIBTL	Test2	78.47	0.785	67.39	89.55	0.431
CAPTP	Test2	82.95	0.901	73.91	91.98	0.525

Considering the potential overlap between the Test1 dataset and the training set of the ToxIBTL, we took precautionary measures to ensure the fairness of our comparative evaluation. To this end, we carefully identified and removed any samples from Test1 that were present in the ToxIBTL training dataset. The resultant dataset Test2 serves as a refined testing ground to reassess the models, and the results are also presented in Table 2 and Fig. 2. Upon reevaluation with the Test2 dataset, our CAPTP model outperforms ToxIBTL across all evaluated metrics, with increases of 4.5% in BACC, 11.6% in AUC, 6.5% in SN, 2.4% in SP, and 9.4% in MCC. These results further confirm the robustness of our model and highlight its potential as a reliable tool for predicting peptide toxicity in novel samples.

### 3.3 Comparative analysis of CAPTP with classical feature-based models

To evaluate the effectiveness of CAPTP, we also used our new dataset to benchmark its performance against various classical machine learning models trained with different handcrafted feature methods. For this comparative analysis, we selected five representative protein statistical features for comparison and analysis: amino acid composition (AAC), grouped amino acid composition (GAAC), quasi-sequence-order descriptors (QSOrder), pseudo-amino acid composition (PAAC), and the normalized Moreau-Broto (NMBroto). All the features mentioned above were extracted using the iLearn package (Chen *et al.* 2021). We adopted several classic machine learning algorithms, including logistic regression, support vector machine, random forest, and light gradient boosting machine (LGBM), which are widely used in biological sequence analysis and prediction tasks. Hyperparameter settings through grid search for these classifiers are detailed in Supplementary Table S3, and the results for the corresponding models are summarized in Supplementary Table S4.

**Figure 3.**
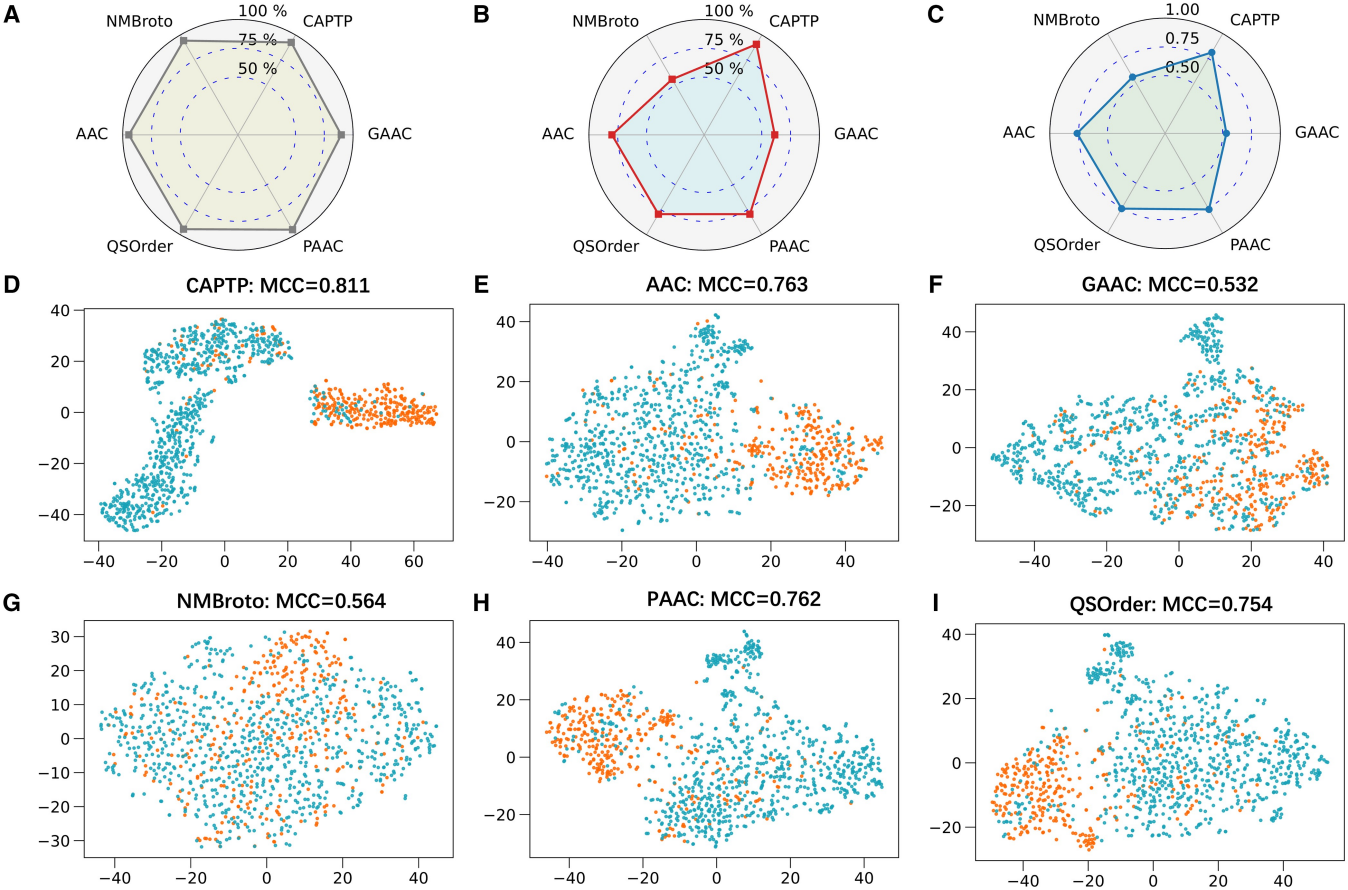
Comparison between CAPTP and five handcrafted feature encoding methods, namely, the AAC, GAAC, NMBroto, PAAC, and QSOrder. (A–C) are comparisons of SP, SN, and MCC values of different methods, respectively. (D–I) are the feature visualization results of each method.

The results indicate a consistent pattern across different features and machine learning algorithms. Notably, the LGBM classifier consistently outperforms the other algorithms across various features. To visually depict this trend, we employed radar charts to compare the SP, SN, and MCC of five LGBM models trained on different protein features with our CAPTP model (Fig. 3A–C). The SP scores for the five features are comparable to those of our model, with our model exhibiting a slight superiority. More notably, our model demonstrates an 10.6%–35.0% greater SN score. This highlights our model’s capacity to accurately identify positive cases, which is essential in toxicity prediction. When evaluating overall performance using the MCC, NMBroto and GAAC scores are notably lower, approximately 0.55, indicating their limited efficacy in the current predictive context. Models such as the QSOrder, PACC, and AAC show similar performance levels, with MCC values of approximately 0.75. However, these values are substantially lower than our model’s MCC of 0.81. These findings further demonstrate the superior predictive accuracy and robustness of our method compared with diverse handcrafted protein features for peptide toxicity prediction.

To enhance the interpretability and further demonstrate the performance of CAPTP, we utilized the dimensionality reduction technique t-distributed stochastic neighbor embedding (t-SNE) (Van der Maaten and Hinton 2008) to visualize the feature representation vectors of peptide sequences. Both t-SNE visualizations derived from five traditional encoding methodologies and those learned by CAPTP are illustrated in Fig. 3D–I. It is evident that our model distinctly segregates the two class samples within its learned feature space, whereas in feature spaces of handcrafted features, a significant proportion of samples appear mixed. This visual distinction is in good agreement with our quantitative findings, as reflected by the MCC score. These findings further confirm the superiority of our model in extracting more effective feature representations for peptide toxicity prediction, compared to traditional handcrafted feature encoding methods.

### 3.4 Exploring the sequential characteristics of peptide toxicity

We further explored the sequential characteristics of peptide toxicity from a computational perspective using the *in silico* mutagenesis (ISM) technique (Nair *et al.* 2022). ISM is a widely used method for determining how each character in an input sequence influences the prediction of a model. Specifically, each reference amino acid in the peptide was mutated to each of the 19 other possible amino acids to assess the absolute change in the predicted probability (referred to as the ISM score).

**Figure 4.**
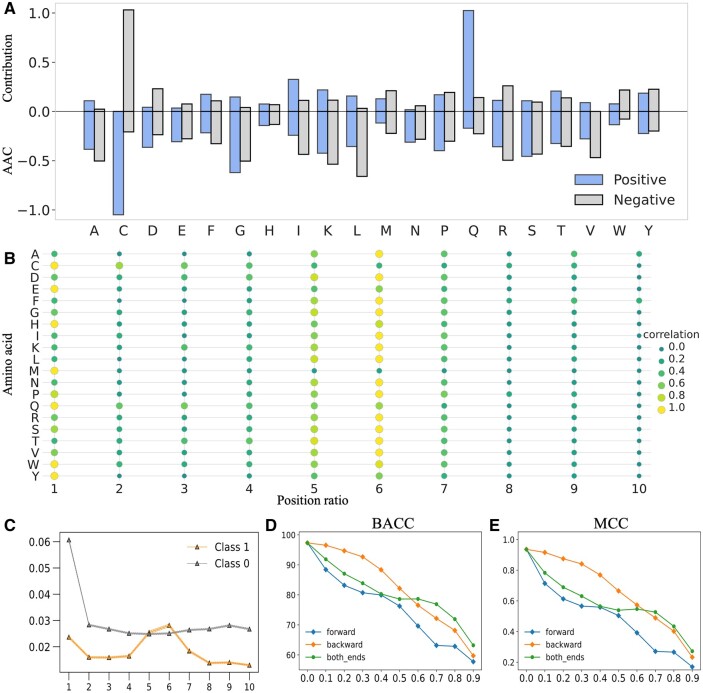
Exploring the sequential characteristics and regional importance of peptide toxicity. (A) shows the comparison of contribution scores for each amino acid from the ISM experiment with the AAC of the respective amino acids. (B) shows the correlation map between the amino acid and the position ratio of ToxPs. (C) shows the mean and variance of the absolute changes in ISM predictive scores across different position ratios. (D and E) show the BACC and MCC results of the clipping experiments.

First, we evaluated the influence of each amino acid from the ISM experiments across the whole dataset. For each sample, we obtained scores of 20 amino acids by summing up scores from every individual position in the sequence based on the ISM outcome. Subsequently, we calculated the contributions of each amino acid to the ToxPs and non-ToxPs separately by taking the mean of these scores for the positive and negative samples, respectively. Figure 4A shows the contributions of 20 amino acids within the ToxPs dataset and non-ToxPs dataset, with the AAC as the reference for the analysis. For most amino acids, the relative contribution to the prediction score is similar to that of the AAC. Notably, while the AAC suggests a minor role for the amino acid Q (glutamine) in the ToxPs dataset, our model assigns significant predictive importance to this amino acid. This indicates that our model could mine features and sequential patterns that cannot be extracted by traditional statistical methods.

We proceeded to explore the relationship between sequence region and peptide toxicity using the results from the ISM experiment. To standardize the sequences to a fixed length, we employed a binning approach, dividing each sequence into 10 segments. In this context, each segment is referred to as a “position ratio.” Figure 4B displays a correlation map between the amino acids and the position ratios of the ToxPs. The correlation values vary significantly across different positions in the sequence. Position 6 shows the highest average correlation, followed by positions 1 and 5. This pattern indicates that certain positions within peptide sequences may be more critical for toxicity attributes than others. Some positions show higher correlation values for certain amino acids; for instance, A (Alanine) has a significant functional impact on the central region, whereas C (Cysteine) predominantly affects the head region. Notably, position 1 exhibits high correlations for half of the amino acids, while position 6 exhibits high correlations for almost all the amino acids except for C (Cysteine) and M (Methionine). Understanding these position-specific correlations is crucial for designing peptides with controlled or reduced toxicity, and all the above observations are meaningful for future peptide design. To visualize the importance of different regions more intuitively, we calculated the mean and variance for each position, as shown in Fig. 4C. For ToxPs, the importance scores of the head region and center region are significantly greater than those of the other positions. For non-ToxPs, there is almost no obvious difference in the importance of most positions except for the head region.

To further validate the above observations, a sequence truncation experiment was conducted on the entire dataset. In this experiment, sequences were truncated in three ways: from the forward end only, from the backward end only, and simultaneously from both ends. These truncated sequences were subsequently fed into our model to evaluate changes in predictive performance. Figure 4D and E displays the trends of BACC and MCC observed during this process. As we can see, the backward clip is more stable with BACC consistently above 90% up to a clipping ratio of 0.4, suggesting that the tail regions of sequences may not be critically influential on the model’s predictive ability. In contrast, forward clipping is associated with a rapid decline in performance. When the clipping ratio is 0.3, a nearly 20% decrease in BACC is observed compared to the model’s performance without clipping, highlighting the importance of the sequence head region. With simultaneous clipping from both ends, as the truncation ratio reaches 0.4, the performance decline in BACC is similar to the pattern observed in forward clipping. Interestingly, when the clipping ratio is between 0.4 and 0.7, the performance seems to enter a stable stage, and when the clipping ratio is greater than 0.7, the BACC continues to drop sharply. This observation suggests that our model assigns greater importance to both the central region and the head region of the sequence, while considering the region between the head and the center, as well as the second half of the sequence, to be less critical for peptide toxicity. As can be seen from Fig. 4E, the trend of MCC is very similar to that of BACC. Taken together, these results provide additional evidence that peptide toxicity may be associated with the head and central regions of the sequence.

## 4 Conclusion

Employing bioinformatics in the discovery of new bioactive peptides with low toxicity can significantly reduce experimental time and costs. Hence, there is a high demand for the development of fast and accurate predictive models. In this work, we curated a new dataset of toxic and non-toxic peptides, and addressed the sample label inconsistencies present in previous datasets. Based on this dataset, we introduced a novel deep learning model named CAPTP, an end-to-end solution specifically tailored for peptide toxicity prediction. Our model effectively combines the local sensitivity of convolution with the ability of the multi-head attention mechanism to manage long-range dependencies, meeting the key needs of global and local sequence analysis and being better at capturing detailed information than handcrafted features. To address the data imbalance, we implemented a contrastive learning strategy that significantly enhances the model’s performance by encouraging the learning of robust and discriminative features from limited data. CAPTP outperforms existing methods, providing superior performance while maintaining a balance between sensitivity and specificity. By utilizing ISM interpretation methods, CAPTP can identify the contribution of each amino acid and uncover distinct sequence patterns. Notably, our model reveals that the head region, central region, and certain sequential patterns are closely related to peptide toxicity. The current version of CAPTP, while effective in sequence pattern analysis, does not include structural information, which is crucial for comprehensive functional analysis and downstream applications. Additionally, our model only classifies peptides as toxins or non-toxins without considering their source of origin. These limitations provide prospects and directions for future improvements and developments. Despite these challenges, we remain optimistic about CAPTP’s potential for designing improved and accurate peptide-based therapeutics against various diseases in the future.

## Supplementary Material

btae297_Supplementary_Data
