# Supplementary material for: Integrated convolution and self-attention for improving peptide toxicity prediction
Source: Bioinformatics. 2024 May 2;40(5):btae297. doi: 10.1093/bioinformatics/btae297 (PMC11654579; doi:10.1093/bioinformatics/btae297)
Supplement: btae297_Supplementary_Data [file btae297_supplementary_data.docx]

**Integrated convolution and self-attention for improving peptide toxicity prediction**

Shihu Jiao^1^, Xiucai Ye^1,^*, Tetsuya Sakurai^1^, Quan Zou^2,3^, Ruijun Liu^4,^*

1. Department of Computer Science, University of Tsukuba, Tsukuba 3058577, Japan
2. Institute of Fundamental and Frontier Sciences, University of Electronic Science and Technology of China, Chengdu 610054, China
3. Yangtze Delta Region Institute (Quzhou), University of Electronic Science and Technology of China, Quzhou 324000, China
4. School of Software, Beihang University, Beijing 100191, China

*Corresponding author: Xiucai Ye: [yexiucai@cs.tsukuba.ac.jp](mailto:yexiucai@cs.tsukuba.ac.jp); Ruijun Liu: liuruijun@buaa.edu.cn

**Contents**

**1 Supporting Figures**

Figure S1. Data processing workflow P2

Figure S2. Amino acid distributions and length distributions of toxic and non-toxic peptides in the training set and testing set P2

**2 Supporting Tables**

Table S1. Label distribution of inconsistently labeled samples in different datasets

P3-P5

Table S2. Hyperparameters values of CAPTP P5

Table S3. Hyperparameters search range for four traditional classifiers P5

Table S4. Comparative performance of CAPTP and classic machine learning models P5-P6


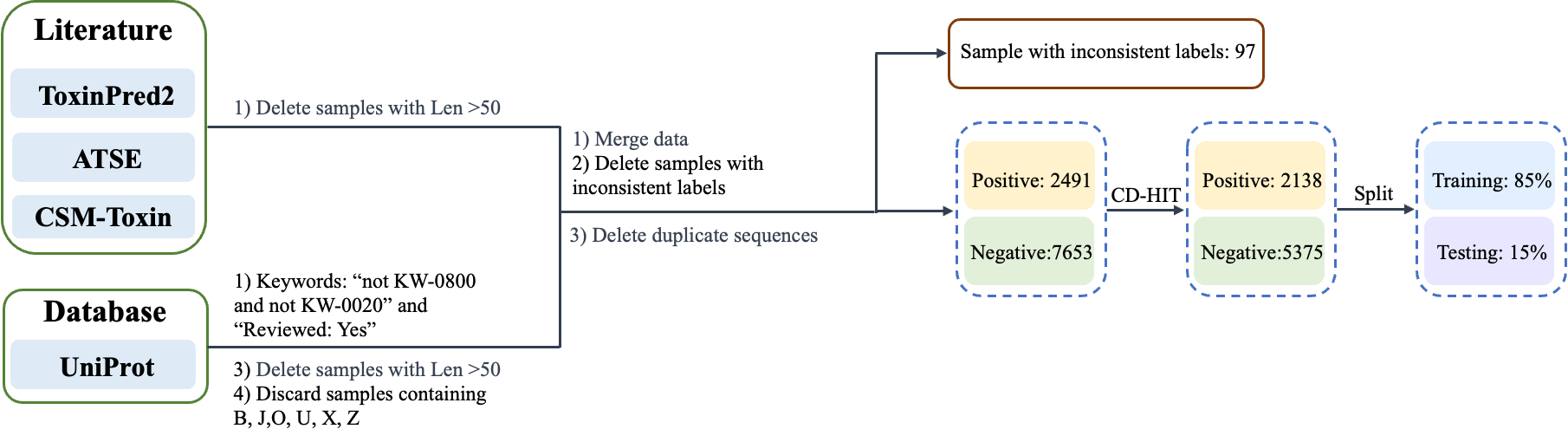


Figure S1. Data processing workflow.

Figure S2. Amino acid distributions and length distributions of toxic and non-toxic peptides in the training set and testing set.

1. **Supporting Tables**

Table S1. Label distribution of inconsistently labeled samples in different datasets. The last column shows the predictions from our model CAPTP, with labels indicating toxic peptides (1), non-toxic peptides (0), or data not available (NA).

| Peptide | CSM-Toxin | ToxinPred2 | ATSE | UniProt | CAPTP_prediction |
| --- | --- | --- | --- | --- | --- |
| GSCLELGKYCDGSKDDCQCCRDNAYCGCDIFGYNWE | 0 | 1 | NA | 0 | 1 |
| MLTKYALVAIIVLCCTVLGFTLMVGDSLCELSIRERGMEFKAVLAYESKK | NA | 1 | NA | 0 | 1 |
| RVCMGKSQHHSFPCISDRLCSNECVKEDGGWTAGYCHLRYCRCQKAC | NA | 1 | 0 | 0 | 1 |
| KASSSAPKGWTHHGSRFTFHRGSM | NA | NA | 0 | NA | 0 |
| RTCQSQSHKFKGACFSDTNCDSVCRTENFPRGQCNQHHVERKCYCERDC | 0 | 1 | NA | 0 | 1 |
| KVCRQRSAGFKGPCVSDKNCAQVCLQEGWGGGNCDGPFRRCKCIRQC | NA | 1 | 0 | 0 | 0 |
| RDCKSDSHKFHGACFSDTNCANVCQTEGFTRGKCDGIHCHCIKDC | NA | 1 | NA | 0 | 1 |
| GGCINHGQPCDGDKNDCQCCRDNGYCNCDGIFGLKWNCKC | 0 | 1 | NA | 0 | 1 |
| MKIRCFCIVLIVSGALLTEVNNNRSLSGDNLLVVNNLQSSK | 0 | 1 | NA | 0 | 0 |
| RECRSQSKQFVGLCVSDTNCASVCLTEHFPGGKCDGYRRCFCTKDC | NA | 1 | 0 | 0 | 1 |
| MPQRTFLMMLIVICVTILCFVWMVRDSLCGLRLQQGNTVLVATLAYEVKR | NA | 1 | NA | 0 | 0 |
| RICRRRSAGFKGPCVSNKNCAQVCMQEGWGGGNCDGPLRRCKCMRRC | 0 | 1 | 0 | 0 | 1 |
| RVCESQSHGFKGACTGDHNCALVCRNEGFSGGNCRGFRRRCFCTLKC | NA | 1 | NA | 0 | 1 |
| MKTLLVFLLLAILVAVLIGNIQVEACKDLTECDTFDICVKGRCYPSTLG | NA | 1 | NA | 0 | 1 |
| LCNERPSQTWSGNCGNTAHCDKQCQDWEKASHGACHKRENHWKCFCYFNC | 0 | 1 | 0 | 0 | 1 |
| RVCRRRSAGFKGLCMSDHNCAQVCLQEGWGGGNCDGVMRQCKCIRQC | NA | 1 | NA | 0 | 1 |
| MLTKYALVAVIVLCLTVPGFTLLVGDSLCEFTVKERNIEFRAVLAYEPKK | NA | 1 | NA | 0 | 1 |
| RECRSESKKFVGLCVSDTNCASVCLTERFPGGKCDGYRRCFCTKDC | 0 | 1 | NA | 0 | 1 |
| GIGAILKVLATGLPTLISWIKNKRKQ | NA | NA | 0 | NA | 0 |
| MPQKYRLLSLIVICFTLLFFTWMIRDSLCELHIKQESYELAAFLACKLKE | 0 | 1 | NA | 0 | 0 |
| ATCDALSFSSKWLTVNHSACAIHCLTKGYKGGRCVNTICNCRN | 0 | 1 | 0 | 0 | 1 |
| LLGRCKVKSNRFHGPCLTDTHCSTVCRGEGYKGGDCHGLRRRCMCLC | 0 | 1 | 0 | 0 | 1 |
| ELCEKASKTWSGNCGNTGHCDNQCKSWEGAAHGACHVRNGKHMCFCYFNC | 0 | 1 | NA | 0 | 0 |
| LLGRCKVKSNRFNGPCLTDTHCSTVCRGEGYKGGDCHGLRRRCMCLC | NA | 1 | NA | 0 | 1 |
| MTLAQFAMIFWHDLAAPILAGIITAAIVSWWRNRK | 0 | 1 | NA | 0 | 1 |
| KTCMTKKEGWGRCLIDTTCAHSCRKYGYMGGKCQGITRRCYCLLNC | 0 | 1 | 0 | 0 | 1 |
| MLTKYALVAVIVLCLTVLGFTLLAGDSLCEFTVKERNIEFRAVLAYEPKK | NA | 1 | NA | 0 | 1 |
| KTCEHLADTYRGVCFTNASCDDHCKNKAHLISGTCHNWKCFCTQNC | 0 | 1 | 0 | 0 | 1 |
| RECQSQSHRYKGACVHDTNCASVCQTEGFSGGKCVGFRGRCFCTKHC | NA | 1 | NA | 0 | 1 |
| VTCDLLSFEAKGFAANHSICAAHCLVIGRKGGACQNGVCVCRN | NA | 1 | NA | 0 | 1 |
| MLTKYALVAVIVLCLTVLGFTLLVGDSLCEFTVKERNIEFKAVLAYEPKK | NA | 1 | NA | 0 | 0 |
| RTCESQSHKFKGPCFSDSNCATVCRTENFPRGQCNQHHVERKCYCERSC | NA | 1 | 0 | 0 | 0 |
| MRTLLVFLLLAILVAVLIGNVQVEACKQGIDCKYPRGCIEGVCEPLYG | NA | 1 | NA | 0 | 0 |
| VTCDLLSFEAKGFAANHSICAAHCLAIGRKGGSCQNGVCVCRN | NA | 1 | NA | 0 | 1 |
| VTCDLLSLQIKGIAINDSACAAHCLAMRRKGGSCKQGVCVCRN | NA | 1 | NA | 0 | 1 |
| MKTLLVFLLLAILVAVLIGNIQVEACKDLTECSAGNRCMHGRCLPMLG | NA | 1 | NA | 0 | 1 |
| MKTLLVFLLLAILVAVLIGNSQVEACNDNAQCGPLGACIMGYFLPIG | 0 | 1 | NA | 0 | 0 |
| GFGCPWNRYQCHSHCRSIGRLGGYCAGSLRLTCTCYRS | 0 | 1 | NA | 0 | 1 |
| ACNFQSCWATCQAQHSIYFRRAFCDRSQCKCVFVRG | 0 | 1 | 0 | 0 | 1 |
| RDCRSQSKTFVGLCVSDTNCASVCLTEHFPGGKCDGYRRCFCTKDC | NA | 1 | NA | 0 | 1 |
| YVSCLFRGARCRVYSGRSCCFGYYCRRDFPGSIFGTCSRRNF | 0 | 1 | NA | 0 | 0 |
| DKLIGSCVWGATNYTSDCNAECKRRGYKGGHCGSFWNVNCWCEE | NA | 1 | NA | 0 | 1 |
| ATCDLLSGIGVQHSACALHCVFRGNRGGYCTGKGICVCRN | NA | 1 | 0 | 0 | 1 |
| MVRARRGCGCPLNQGACHRHCKSIGRRGGYCAGFLKQTCTCYRN | NA | 1 | NA | 0 | 0 |
| NLCERASLTWTGNCGNTGHCDTQCRNWESAKHGACHKRGNWKCFCYFNC | NA | 1 | 0 | 0 | 1 |
| MPQRTFLMMLIVVCVTILCFVWMVRDSLCGFRIEQGNTVLVATLAYEVKR | 0 | 1 | NA | 0 | 0 |
| RVCRRRSAGFKGLCMSDHNCAQVCLQEGWGGGNCDGVIRQCKCIRQC | NA | 1 | NA | 0 | 1 |
| GFGCPLDQMQCHRHCQTITGRSGGYCSGPLKLTCTCYR | 0 | 1 | NA | 0 | 1 |
| MKTLLVFLLLAILVAVFIGNAQVEACKNHTDCLSGICLKGFCMPAFG | 0 | 1 | NA | 0 | 1 |
| ATCDLLSGTGANHSACAAHCLLRGNRGGYCNGKAVCVCRN | NA | 1 | 0 | 0 | 1 |
| GFGCPGDAYQCSEHCRALGGGRTGGYCAGPWYLGHPTCTCSF | 0 | 1 | NA | 0 | 1 |
| MRTLLVFLLLAILVAVLIGNVQVEACKEVDECGDMFVCIEGICEPLIG | NA | 1 | NA | 0 | 0 |
| INFGAIKAILKKWGKKLVEYALKHKDLYAPYIKKHLCEKL | 1 | 1 | 0 | NA | 1 |
| KICRRRSAGFKGPCMSNKNCAQVCQQEGWGGGNCDGPFRRCKCIRQC | NA | 1 | 0 | 0 | 1 |
| MKQHKAMIVALIVICITAVVAALVTRKDLCEVHIRTGQTEVAVFTAYESE | NA | 1 | NA | 0 | 0 |
| AICKKPSKFFKGACGRDADCEKACDQENWPGGVCVPFLRCECQRSC | 0 | 1 | 0 | 0 | 1 |
| GFGCPFNQGACHRHCRSIRRRGGYCAGLFKQTCTCYR | NA | 1 | 0 | 0 | 1 |
| RTCQSQSHKFKGACFSDTNCASVCRTENFPRGQCNQHHVERKCYCERDC | NA | 1 | NA | 0 | 1 |
| GFMDLIKKAGGWLKKKGPALIKAALQE | 1 | NA | 0 | NA | 0 |
| DKLIGSCVWGAVNYTSNCNAECKRRGYKGGHCGSFANVNCWCET | NA | 1 | 0 | 0 | 1 |
| FTCDVLGFEIAGTKLNSAACGAHCLALGRRGGYCNSKSVCVCR | 0 | 1 | 0 | 0 | 1 |
| RECQSQSHRYKGACVHDTNCASVCQTEGFSGGKCVGFRGRCFCTKAC | NA | 1 | NA | 0 | 1 |
| KFCEKPSGTWSGVCGNSGACKDQCIRLEGAKHGSCNYKPPAHRCICYYEC | NA | 1 | NA | 0 | 0 |
| GWKDWAKKAGGWLKKKGPGMAKAALKAAMQ | 1 | NA | 0 | NA | 1 |
| MQIVEAIYEDGVLKLLKNLKLKEHSKVIIKVIDEEEIEKILDSRDY | 0 | 1 | NA | 0 | 0 |
| MRTLLVFLLLAIFVAVLIGNVQVEAACKEYWECGAFLFCIEGICVPMIG | 0 | 1 | NA | 0 | 0 |
| QGGWPRPGPEIPP | 0 | NA | 1 | 0 | 1 |
| KDCKRESNTFPGICITKPPCRKACIREKFTDGHCSKILRRCLCTKPC | NA | 1 | NA | 0 | 1 |
| RDCESDSHKFHGACFSDTNCANVCQTEGFTAGKCVGVQRHCHCTKDC | 0 | 1 | NA | 0 | 1 |
| FIGTALGIASAIPAIVKLFK | 1 | NA | 0 | NA | 0 |
| MKHNPLVVCLLIICITILTFTLLTRQTLYELRFRDGDKEVAALMACTSR | 0 | 1 | NA | 0 | 0 |
| MRTLLVFLLLTILVAVLIGNNQVEACTNNADCHGLGHCYRGTCFPVMG | 0 | 1 | NA | 0 | 1 |
| GIWGTALKWGVKLLPKLVGMAQTKKQ | 1 | NA | 0 | NA | 0 |
| KTCENLSGTFKGPCIPDGNCNKHCRNNEHLLSGRCRDDFRCWCTNRC | 0 | 1 | 0 | 0 | 1 |
| MKTLLVFLLLAILVAVLIGNIQVEACKDFNECTDFDICVKGRCYPSMLG | 0 | 1 | NA | 0 | 1 |
| MRTLLVFLLLAILVAVLIGNIQVEACKDLTECSPYKLCIKGICERMIG | NA | 1 | NA | 0 | 1 |
| VTCDLLSFEAKGFAANHSLCAAHCLAIGRRGGSCERGVCICRR | NA | 1 | 0 | 0 | 1 |
| ATCDLLSGTGIKHSACAAHCLLRGNRGGYCNGRAICVCRN | NA | 1 | 0 | 0 | 1 |
| GFGCPLNQGACHRHCRSIRRRGGYCAGFFKQTCTCYRN | NA | 1 | 0 | 0 | 0 |
| RDCTSQSHKFVGLCLSDRNCASVCLTEYFTGGKCDHRRCVCTKGC | NA | 1 | 0 | 0 | 1 |
| ATCRKPSMYFSGACFSDTNCQKACNREDWPNGKCLVGFKCECQRPC | 0 | 1 | 0 | 0 | 1 |
| MTLAQFAMTFWHDLAAPILAGIITAAIVGWWRNRK | NA | 1 | NA | 0 | 0 |
| MKVRCFCVVLLVSGTLCLHADRSYPGNSVPVTLNVQSR | 0 | 1 | NA | 0 | 0 |
| RVCMGKSQHHSFPCISDRLCSNECVKEEGGWTAGYCHLRYCRCQKAC | 0 | 1 | NA | 0 | 1 |
| MTFAELGMAFWHDLAAPVIAGILASMIVNWLNKRK | 0 | 1 | NA | 0 | 0 |
| QKLCERPSGTWSGVCGNNNACKNQCINLEKARHGSCNYVFPAHK | NA | 1 | NA | 0 | 0 |
| MTVYESLMIMINFGGLILNTVLLIFNIMMIVTSSQKKK | 0 | 1 | NA | 0 | 0 |
| DKLIGSCVWGAVNYTSDCNGECKRRGYKGGHCGSFANVNCWCET | NA | 1 | NA | 0 | 1 |
| RVCMGKSAGFKGLCMRDQNCAQVCLQEGWGGGNCDGVMRQCKCIRQCW | 0 | 1 | 0 | 0 | 1 |
| KFCEKPSGTWSGVCGNSGACKDQCIRLEGAKHGSCNYKLPAHRCICYYEC | NA | 1 | NA | 0 | 0 |
| RYCPRNPEACYNYCLRTGRPGGYCGGRSRITCFCFR | 0 | 1 | NA | 0 | 0 |
| GIFSSRKCKTPSKTFKGYCTRDSNCDTSCRYEGYPAGD | NA | 1 | 0 | 0 | 1 |
| GVGCSSGCHKVGGQCRCG | 1 | NA | 0 | NA | 1 |
| ATCDILSFQSQWVTPNHAGCALHCVIKGYKGGQCKITVCHCRR | 0 | 1 | NA | 0 | 0 |
| MLTKYALAAVIVLCLTVLGFTLLVGDSLCEFTVKERNIEFKAVLAYEPKK | 0 | 1 | NA | 0 | 0 |
| RPPGFSPFRIAPASSL | 1 | NA | 0 | NA | 1 |
| RVCRRRSAGFKGVCMSDHNCAQVCLQEGYGGGNCDGIMRQCKCIRQC | NA | 1 | 0 | 0 | 0 |

Table S2. Hyperparameters values of CAPTP.

| **Hyperparameters** | **Values** |
| --- | --- |
| Batch size | 512 |
| Learning rate | 0.00003 |
| Dimension of adaptive embedding | 512 |
| kernel size in convolutional modulation | 3 |
| Dimension of K and V | 64 |
| Feedforward neural network | 2048 |
| Number of heads of multi-head self-attention | 8 |
| Number of layers of multi-head self-attention | 1 |
| Linear layers in projection layer | [512, 128, 64] |
| Dropout in projection layer | 0.5 |

Table S3. Hyperparameters search range for four traditional classifiers.

| **Method** | Parameter***** | **Tested values** |
| --- | --- | --- |
| LR | C | [0.01, 0.1, 10, 100] |
| RF | n_estimators | 100–500 with an interval of 100 |
| SVM | C | [2^-5^–2^15^] in log_2_ steps |
|  | gamma | [2^-15^–2^-5^] in log_2_ steps |
| LGBM | n_estimator | 100-500 with an interval of 100 |

^*^Parameter name in the Scikit-learn implementation.

Table S4. Comparative performance of CAPTP and classic machine learning models.

| **Algorithm** | **Feature** | **BACC (%)** | **AUC** | **SN (%)** | **SP (%)** | **MCC** |
| --- | --- | --- | --- | --- | --- | --- |
| LR | AAC | 82.49 | 0.888 | 70.94 | 94.05 | 0.682 |
|  | GAAC | 68.28 | 0.824 | 44.38 | 92.18 | 0.427 |
|  | PAAC | 82.46 | 0.899 | 71.25 | 93.67 | 0.678 |
|  | NMBroto | 68.53 | 0.790 | 44.38 | 92.68 | 0.436 |
|  | QSOrder | 82.56 | 0.903 | 71.56 | 93.55 | 0.679 |
| RF | AAC | 87.02 | 0.950 | 78.75 | 95.29 | 0.764 |
|  | GAAC | 74.10 | 0.858 | 59.38 | 88.83 | 0.503 |
|  | PAAC | 86.24 | 0.940 | 77.19 | 95.29 | 0.752 |
|  | NMBroto | 71.99 | 0.835 | 48.44 | 95.53 | 0.528 |
|  | QSOrder | 85.77 | 0.941 | 76.25 | 95.29 | 0.745 |
| SVM | AAC | 86.65 | 0.925 | 78.75 | 94.54 | 0.751 |
|  | GAAC | 72.10 | 0.818 | 53.13 | 91.07 | 0.485 |
|  | PAAC | 86.43 | 0.924 | 77.81 | 95.04 | 0.752 |
|  | NMBroto | 73.67 | 0.853 | 51.56 | 95.78 | 0.560 |
|  | QSOrder | 86.65 | 0.934 | 78.75 | 94.54 | 0.751 |
| LGBM | AAC | 87.33 | 0.946 | 80.00 | 94.67 | 0.763 |
|  | GAAC | 75.48 | 0.856 | 61.25 | 89.70 | 0.532 |
|  | PAAC | 87.14 | 0.937 | 79.38 | 94.91 | 0.762 |
|  | NMBroto | 74.96 | 0.842 | 55.63 | 94.29 | 0.564 |
|  | QSOrder | 86.90 | 0.945 | 79.38 | 94.42 | 0.754 |
| CAPTP | | 91.59 | 0.959 | 90.63 | 92.56 | 0.811 |

LR: Logistic Regression, RF: Random Forest, SVM: Support Vector Machine, LGBM: Light Gradient Boosting Machine.
